# Magnesium sulfate treatment for juvenile ferrets following induction of hydrocephalus with kaolin

**DOI:** 10.1186/s12987-016-0031-4

**Published:** 2016-04-27

**Authors:** Domenico L. Di Curzio, Emily Turner-Brannen, Xiaoyan Mao, Marc R. Del Bigio

**Affiliations:** Department of Human Anatomy & Cell Science, University of Manitoba, Winnipeg, MB Canada; Children’s Hospital Research Institute of Manitoba, Winnipeg, MB Canada; Department of Pathology, University of Manitoba Brodie 401-727 McDermot Avenue, Winnipeg, MB R3E 3P5 Canada

**Keywords:** Hydrocephalus, Ferret, Kaolin, Magnesium sulfate, Brain

## Abstract

**Background:**

Previous work with 3-week hydrocephalic rats showed that white matter damage could be reduced by the calcium channel antagonist magnesium sulfate (MgSO_4_). We hypothesized that MgSO_4_ therapy would improve outcomes in ferrets with hydrocephalus induced with kaolin at 15 days.

**Methods:**

MRI was performed at 29 days to assess ventricle size and stratify ferrets to treatment conditions. Beginning at 31 days age, they were treated daily for 14 days with MgSO_4_ (9 mM/kg/day) or sham saline therapy, and then imaged again before sacrifice. Behavior was examined thrice weekly. Histological and biochemical ELISA and myelin enzyme activity assays were performed at 46 days age.

**Results:**

Hydrocephalic ferrets exhibited some differences in weight and behavior between treatment groups. Those receiving MgSO_4_ weighed less, were more lethargic, and displayed reduced activity compared to those receiving saline injections. Hydrocephalic ferrets developed ventriculomegaly, which was not modified by MgSO_4_ treatment. Histological examination showed destruction of periventricular white matter. Glial fibrillary acidic protein content, myelin basic protein content, and myelin enzyme activity did not differ significantly between treatment groups.

**Conclusion:**

The hydrocephalus-associated disturbances in juvenile ferret brains are not ameliorated by MgSO_4_ treatment, and lethargy is a significant side effect.

## Background

Hydrocephalus is a common neurological condition where cerebrospinal fluid (CSF) flow dynamics are altered, leading to enlargement of ventricular cavities in the brain. The histopathologic consequences of hydrocephalus depend on the age of onset, rate of ventricular enlargement, and the etiology [1]. The brain damage induced by hydrocephalus is multifactorial with mechanical factors leading to primary destruction of periventricular axons due to gradual physical stretching and compression, accumulation of metabolic waste products in the CSF, and ischemic changes causing decreased white matter blood flow that contributes to axonal and oligodendroglial damage [2, 3]. Elevated calcium (Ca^2+^) coincides with increased white matter content of calpain I, along with heightened immunoreactivity in periventricular axons exhibited by young hydrocephalic rats with appreciable axonal damage, which suggest that calcium-mediated proteolysis may be associated with axonal cytoskeletal damage found in hydrocephalus [4].

Magnesium (Mg^2+^) is a calcium channel antagonist; extracellular Mg^2+^ antagonizes Ca^2+^ influx by blocking voltage and receptor-mediated calcium channels along with NMDA channel receptors in a voltage-dependent manner [5, 6]. Intracellular Mg^2+^ is regulated by at least five distinct transporters [7]. Peripherally administered Mg^2+^ enters brain tissue of rabbits and cats, albeit to a lesser magnitude than muscle [8]. Magnesium sulfate (MgSO_4_) administration by bolus or short-term infusion (e.g. 3 days) has shown short-term (hours to <7 days) outcome improvements in animal models of experimental spinal cord injury [9], reversible focal cerebral ischemia [10], kainate induced neuron degeneration [11], hypoxic-ischemic brain damage [12, 13], and traumatic brain injury [14–16]. Few experiments have looked at longer-term benefits of magnesium. A bolus of magnesium chloride after fluid percussion brain injury in adult rats was associated with improved motor outcome at 4 weeks [17].

Clinically, MgSO_4_ has been used to suppress uterine contractions during premature labor and to reduce convulsions in pregnant women with preeclampsia [18]. In these circumstances, there is an association between maternal receipt of MgSO_4_ and reduced risk of cerebral palsy in the offspring; this suggests that it could be neuroprotective in preterm birth although the benefit in term birth is much less clear [19, 20]. In infants with hypoxic-ischemic encephalopathy, the composite results of five clinical trials indicate a significant reduction in the unfavorable short-term outcome but a trend to increased mortality overall [21]. If effective, its protective mechanism remains unclear. MgSO_4_ is a vasodilator that might improve cerebral circulation [22]. It can also reduce monocyte-mediated proinflammatory cytokine production and increase intracellular magnesium levels, which may be beneficial in decreasing inflammation [23].

Pharmacologic intervention might be a useful supplement to surgical shunts for the management of hydrocephalus [24]. We previously showed that MgSO_4_ was beneficial in hydrocephalic rats [25]. Three-week old rats received kaolin injections into the cisterna magna to induce hydrocephalus; after 2 weeks, they were treated with parenteral administration of MgSO_4_ for 2 more weeks. There was reduced reactive astrogliosis in the frontal cerebrum and improved gait performance on an accelerating rotating cylinder task. Despite these benefits, research with rodents is limiting because they have a small volume of white matter, which is the main region of damage in human brains. Moreover, stroke research has provided a valuable lesson in the major setbacks that can occur when basing clinical treatment on the success of pharmacological studies with rodents; this has prompted the necessity of preclinical treatment efficacy in gyrencephalic brains before proceeding to human trials [26]. Thus, we developed a model of hydrocephalus in 2-week old ferrets using injection of kaolin (aluminum silicate) into the cisterna magna, and we showed similarities to other animal models and the human condition [27]. Ferrets are born with relatively immature brains which develop a complex gyrencephalic morphology that has been studied extensively [28, 29]; consequently they are useful for modeling the neurologic disorders of human fetuses and infants [30]. Our overall goal is to develop a pharmacologic intervention that could be used to mitigate brain damage in the period prior to definitive shunt therapy. We hypothesized that MgSO_4_ treatment would lead to behavioral, structural, and/or biochemical improvements in juvenile ferrets with experimental kaolin-induced hydrocephalus.

## Methods

### Animals

Twenty pigmented sable ferret kits (n = 13 males and n = 7 females) were obtained from Marshall Farms (North Rose, NY) at postnatal day 7 (P7) in 4 l along with their mothers (jills). The kits stayed with their mothers in enclosed cages until P46. The cages were located in a room kept on a 12:12 h (6 a.m.–6 p.m.) light–dark cycle, and the room temperature and relative humidity were 21–22 **°**C and ~35–45 %, respectively. Food and water were provided ad libitum; the kits started eating solid food around P30. For identification, tattoos were imprinted on their paws. All animals were treated humanely according to the guidelines set forth by the Canadian Council on Animal Care. The institutional animal ethics committee approved the experimental protocols (protocol #11-012). All efforts were made to minimize the number of animals used and ensure the least amount of suffering experienced.

### Hydrocephalus induction

Hydrocephalus was induced using kaolin (aluminum silicate; Sigma, St. Louis MO) as described previously [27]. Briefly, kaolin injections were performed on all 4 litters at P15 (5 per litter—total n = 20; weight 69–93 g). They were anesthetized using 2.5 % isoflurane in oxygen, and the dorsum of their necks were shaved and cleansed. Using a 27-gauge needle, 0.2 mL of 20 % sterile kaolin suspension (250 mg/mL in 0.9 % saline) was injected percutaneously into the cisterna magna under aseptic conditions. Animals were monitored during recovery and observed for signs of discomfort and/or neurological impairment and then were otherwise returned to their mothers. Subcutaneous (sc) injections of buprenorphine (0.03 mg/kg) and sterile 0.45 % saline were given every 12 h for 2 days to decrease potential pain and possible dehydration, respectively. They were weighed daily, and those experiencing severe neurologic impairment or weight loss were sacrificed to cease further suffering.

### Magnetic resonance imaging and assignment to treatment groups

The T2-weighted MR images of the brain were obtained using with a 7 Tesla Bruker Biospec/3 MR scanner (Karlsruhe, Germany) as previously described [27]. The first images were attained 2 days post kaolin injections at P17 to confirm successful hydrocephalus induction with kaolin, and then 14 days post kaolin at P29 to examine ventricle size and stratify treatment groups. The areas of the lateral ventricles to cerebrum brain area, third ventricle width to cerebrum width, cerebral aqueduct area to midbrain area, and fourth ventricle area to hindbrain area ratios were measured as previously described [27]. Ferrets were stratified based on ventricle size and assigned listwise to saline (0.9 % NaCl) and MgSO_4_ treatment groups. A third set of MR images was taken after the 14-day drug treatment period at P45, no more than 24 h before euthanasia, to assess ventricle size again.

### Drug preparation and administration

The MgSO_4_ and/or NaCl treatments were administered starting 16 days post-kaolin at P31 (n = 8 for MgSO_4_ and n = 8 for NaCl). All animals were weighed daily, and their weights were used to calculate treatment volumes. Stock solutions of 1.0 M MgSO_4_ and 0.9 M NaCl were prepared in distilled H_2_O at room temperature and stored at 4 °C; the solutions were labeled A and B, and treatments were given blindly based upon a volume drug to body weight formula. They received either a ~9 mM/kg/day dosage of MgSO_4_ or NaCl daily for 14 days, which were administered by sc injections given 3 times per day on weekdays (~3 mM/kg/dose) and 2 times per day on weekends (~4.5 mM/kg/dose) to ensure that all animals received the entire dosage. This calculated dosage was derived from testing different MgSO_4_ concentrations with rodents in a previous study, where 8.2 mM/kg/day was found to be protective [25]. The ferrets received their first injection in the morning before behavioral testing. Their second daily injection was given in the late afternoon, and the third injection was administered near midnight to maintain trough levels. Because of the expense, we could not conduct a dose-escalation study.

### Behavioral testing

Previous work with kaolin-induced hydrocephalic ferrets [27] showed that they differed from controls in a limited number of the behavioral tests previously studied [31]; these were chosen for the current study. Behavioral testing commenced at P10-11 and continued 3 times weekly until P43-44 for all kits (n = 16). Behavior tests were conducted prior to the daily drug administration to minimize the effect of lethargy in the MgSO_4_ recipients. The kits were not exposed to the test situations prior to testing. Open field behavior was assessed using two apparatuses. The first chamber was an enclosed square (44 × 43 × 29 cm) with 15 light beam sensors on each axis to quantitate ambulatory, vertical, and total movements (Opto-Varimex 3; Columbus Instruments International Corp., Columbus, OH, USA). Animals were observed individually for 3 min and tested once per session. The second chamber was a 75 × 75 × 45 cm transparent plastic square, where the kits were videotaped, and motor performance was analyzed for 3 min using HVS Image 2100 Plus Tracking System software (HVS Image Ltd, Twickenham, Middlesex, UK). Quantitative measures were performed after dividing the chamber into 100 (7.5 × 7.5 cm) squares and included the length of path traversed and the number and percent of squares entered. Qualitative observations were also recorded in the second chamber for pivoting, crawling, walking, running, and rearing.

### Sacrifice and brain dissection

Ferrets were euthanized within 24 h of final MRI using isoflurane anesthesia (5 %) followed by carbon dioxide (CO_2_) narcosis and exsanguination by transcardiac perfusion at ~P46 or when humane endpoints were met as described previously [27]. Ferret brains were rapidly removed, photographed, and then split in the parasagittal plane 1 mm from the midline. The right hemisphere was dissected into anterior frontal lobe, dorsal frontal cerebrum, and dorsal parietal cerebrum, which were frozen in liquid nitrogen (N_2_) and stored at −70 °C. The left hemisphere was immersion fixed in cold 3 % buffered paraformaldehyde for several days; then it was sliced in the coronal plane, dehydrated, and embedded in paraffin wax.

### Histology and immunohistochemistry

All paraffin blocks were sectioned coronally (6 μm thickness) and stained with hematoxylin and eosin (H and E). The cerebrum at the level of the anterior horn of the lateral ventricles was stained with solochrome cyanine and counterstained with eosin for visualization of myelin. Sections were immunostained with rabbit polyclonal anti-glial fibrillary acidic protein (GFAP; 1:10,000 dilution; DAKO Z0334; Glostrup, Denmark) to label astrocytes and reactive astrocytes. The primary antibody underwent 1.5 h incubation at room temperature. This was followed by incubation with appropriate biotinylated secondary antibody, followed by reaction with streptavidin-peroxidase, detection with diaminobenzidine (DAB, Sigma D5905), and finally counterstaining with hematoxylin. Negative controls were treated without the primary antibody. Corpus callosum thickness was measured using 100× ocular magnification at the sagittal midline and above the lateral angle of the anterior horn of the lateral ventricle. The second site was chosen because fragmentation of the midline region in some hydrocephalic brains preventing proper measurement of corpus callosum thickness. For comparative purposes, non-hydrocephalic ferrets from our previous experiment [27] were also examined.

### Myelin enzymes and enzyme-linked immunosorbent assays

Frozen dorsal frontal and parietal cerebrum samples were homogenized using a radio immunoprecipitation assay (RIPA) buffer including protease inhibitors phenylmethylsulfonyl fluoride (PMSF) and aprotinin, which likely explain the lower myelin enzyme activities than were obtained previously [27]. Total protein quantification was determined with the Micro BCA (Pierce) Protein Assay kit (Thermo Scientific, Rockford, Illinois, USA). Dorsal frontal cerebrum homogenates were used to quantify myelin basic protein (MBP) and GFAP content by using ELISAs, as previously described in detail [27]. Colorimetric assays were performed in triplicate, and results are shown in μg of MBP or GFAP per gram of protein and averaged per sample. Dorsal parietal cerebrum samples were used to quantify the enzyme activity of UDP-galactose:ceramide galactosyltransferase (CGalT) and glycerylphosphorylcholine phosphocholine phosphodiesterase (GPC-PP) using the artificial substrate p-nitrophenylphosphorylcholine (pNPP). Both are enzymes that are enriched in myelin. They were quantified in triplicate as described previously [32, 33].

### Atomic absorption spectroscopy

Flame atomic absorption spectroscopy (FAAS) was performed to determine Mg levels in anterior forebrain samples as indicated previously [34] except for the following differences. Ferrets were given their last dose of MgSO_4_ or NaCl 35–152 min (mean 68.88 ± 13.46) before sacrifice. Forebrain samples were thawed and weighed then dissolved in 18 M sulfuric acid and 16 M nitric acid over gentle heat. Standards were prepared from a 1000 μg mL^−1^ Mg standard solution (Alfa Aesar 88077) at concentrations of 0.50, 0.40, and 0.20 μg mL^−1^, along with a 1 % nitric acid blank. Mg content was measured using Atomic Absorption Spectrometer AAnalyst 400 with a PerkinElmer Lamp (Intensitron) using a wavelength of 285.21 nm. Measurements were performed in triplicate and averaged to obtain concentrations (μg Mg^2+^ per gram brain tissue).

### Statistical analysis

All data are presented as mean ± SEM, unless otherwise indicated. Quantitative data were analyzed to confirm a normal distribution, and *p* values ≤0.05 were considered statistically significant. Statistical analyses for the behavioral tasks, MRI, and all biochemical analyses were conducted with the juvenile ferrets (n = 15). Data were assessed using ANOVA and two-tailed *t*-tests for behavioral testing, ventricle size, histological data, and biochemical assays to compare MgSO_4_ and NaCl hydrocephalic treatment groups. Qualitative assessments for motor and behavioral development were analyzed separately from quantitative measures. Statistical analyses were conducted using the SPSS 19.0 software program.

## Results

### Mortality

Of the 20 kaolin-injected juvenile ferrets, three were euthanized due to severe neurological deficits within the first 2 days post-injection (P16), one was euthanized on P22 due to severe weight loss and neurological impairment, and one was euthanized on P43 due to persistently low weight and neurological impairment despite only slight ventricle enlargement (this animal was initially assigned to receive MgSO_4_). Two of the remaining ferrets were nonhydrocephalic; these were used for comparative purposes only. The focus of the results is on the hydrocephalic ferrets (n = 13; 8 males and 5 females) that survived the 14-day drug therapy period. All were euthanized within 24 h of the final MRI between postnatal days P45–P46.

### Ventricle size on magnetic resonance imaging

**Fig. 1:**
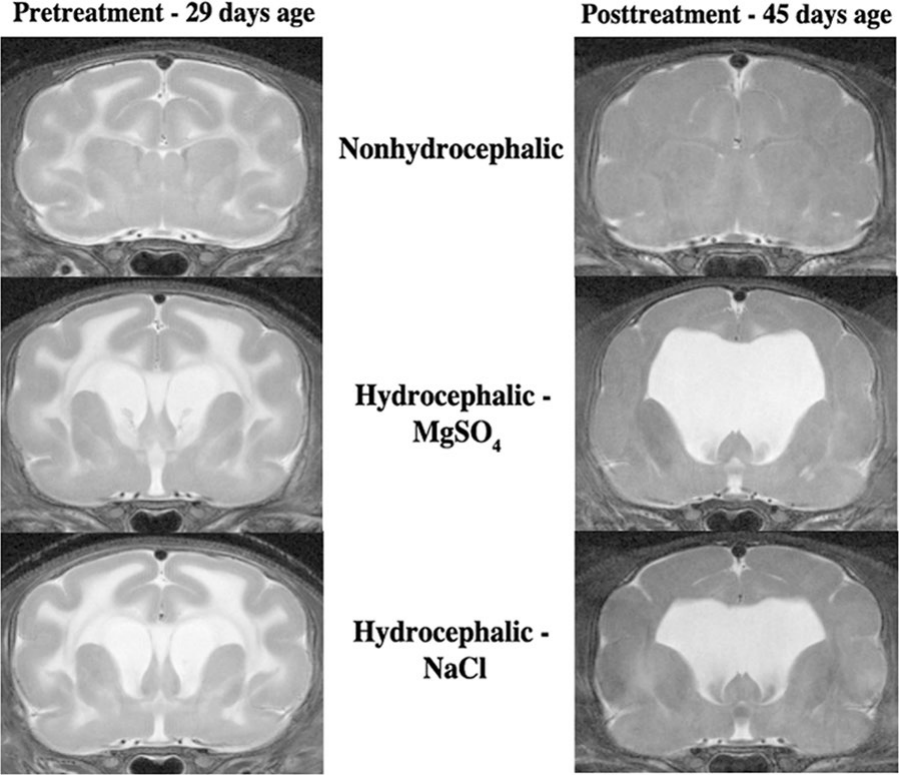
T2-weighted magnetic resonance images showing frontal coronal slices of brains of ferrets without hydrocephalus as well as ferrets that were treated with MgSO_4_ or NaCl between 29 and 45 days age. Progressive ventriculomegaly is evident in these examples of both treatments

**Fig. 2:**
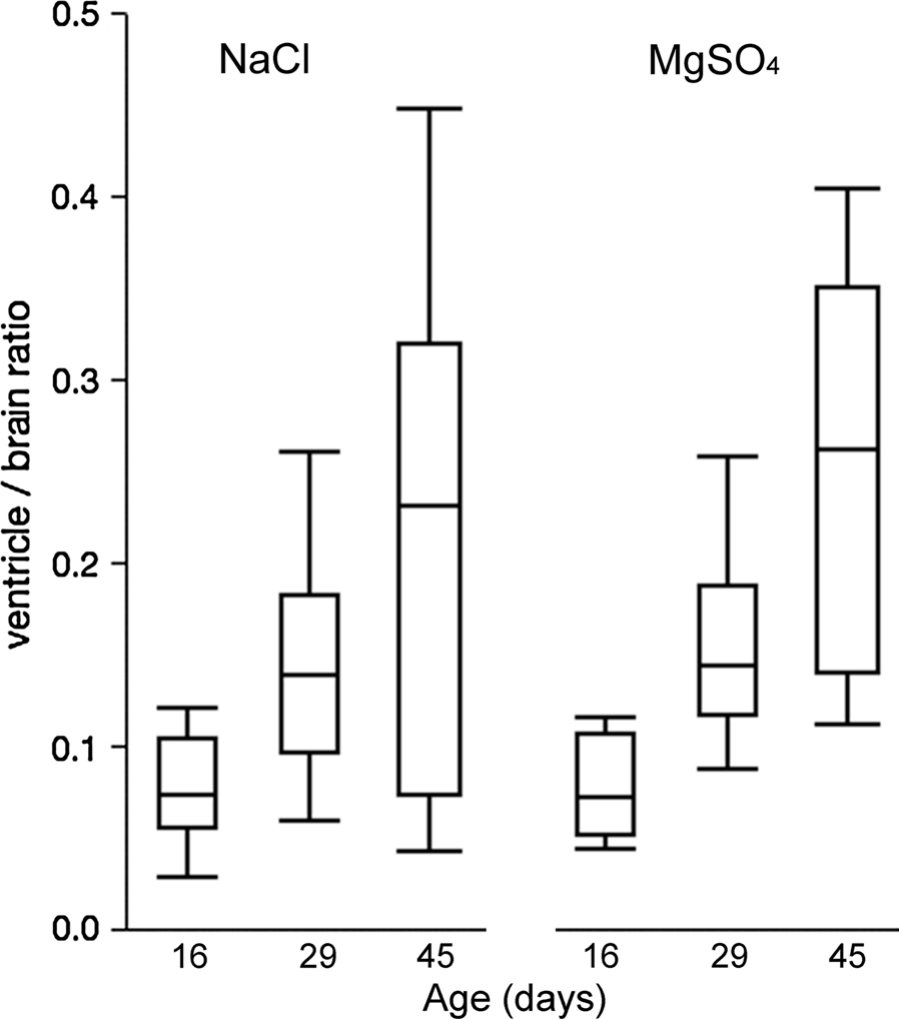
*Box plots *(mean ± standard error of mean) showing cross sectional ratio of the lateral ventricle frontal horn areas to the cerebrum on the magnetic resonance images. The 16-day images were obtained 2 days after kaolin injection, the 29-day images were 14 days after kaolin injection and immediately prior to onset of drug treatment, and the 45-day images were obtained at the end of the treatment period shortly prior to termination (n = 7 for NaCl and n = 6 for MgSO_4_ each at all time points). In comparison to non-hydrocephalic controls (not shown) and previous time points the ventricles enlarged progressively at successive time points (p < 0.05). There were no significant differences between the MgSO_4_ and NaCl treatment groups

Ventricular expansion was noticeable 2 days after kaolin injections, where the lateral ventricles were already significantly larger compared to the nonhydrocephalic animals (*p* < 0.010, *t* test). From this time onward, the hydrocephalic ferrets displayed enhanced signal in the periventricular white matter on the T2-weighted MR images indicating elevated water content. At 14 days post-kaolin injections (P29), MR images showed a range of ventriculomegaly in the hydrocephalic ferrets (Fig. 1). This was particularly evident in the lateral and third ventricles, which were significantly enlarged (both *p* < 0.05, *t* tests). Ferrets were stratified according to ventricle size and alternately assigned to MgSO_4_ or NaCl treatment groups to ensure that there was no significant difference between hydrocephalic groups before therapy. Both hydrocephalic groups displayed further expansion of the ventricles during the therapeutic period, exhibiting significant progressive enlargement of the lateral (Fig. 2; Table 1) and third ventricles (all *p* < 0.05, *t* tests). Comparison of the NaCl- and MgSO_4_-treated ferrets showed no significant differences for any of the ventricle regions (all *p* > 0.05, *t* tests; Table 1).

**Table 1:** Results of magnesium sulfate treatment on hydrocephalic ferrets

	Non-hydrocephalic controls	NaCl-treated hydrocephalus	MgSO_4_-treated hydrocephalus
Sample size	2	7	6
Lateral ventricle area index (P29/pre-treat)	0.012 ± 0.002	0.147 ± 0.024*	0.155 ± 0.023*
Lateral ventricle area index (P45/post-treat)	0.006 ± 0.0005	0.219 ± 0.053*^#^	0.254 ± 0.046*^#^
Percent enlargement ventricles during treatment	–	48.9 ± 23.1	63.9 ± 24.4
Body weight (g) (P29/pre-treat)	148.0 ± 8.0	114.3 ± 4.8*	106.8 ± 9.1*
Body weight (g) (P45/post-treat)	264.5 ± 34.5^#^	288.3 ± 12.8^#^	170.0 ± 31.0*^@#^
Rearing activity (beam breaks per 3 min) (P43/post-treat)	71 ± 16	57 ± 15	22 ± 20*
Ambulatory activity (beam breaks per 3 min) (P43/post-treat)	541 ± 21	460 ± 44	321 ± 71*
Total activity (beam breaks per 3 min) (P43/post-treat)	684 ± 20	584 ± 54	443 ± 89*
Number cells entered—open field (per 3 min) (P43/post-treat)	88 ± 7	115 ± 12	111 ± 15
Distance traveled—open field (m per 3 min) (P43/post-treat)	5.64 ± 0.39	7.58 ± 0.95	7.11 ± 1.08
Medial corpus callosum thickness (μm)	965 ± 95	410 ± 71*	315 ± 97*
Lateral corpus callosum thickness (μm)	665 ± 45	330 ± 50*	245 ± 59*
MBP content frontal cerebrum (μg MBP/g protein)	72.19 ± 12.75	64.25 ± 19.66	56.07 ± 14.15
CGalT activity parietal cerebrum (nM/mg protein/h)	0.029 ± 0.008	0.075 ± 0.024	0.051 ± 0.014
GPC-PP activity parietal cerebrum (nM/mg protein/h)	15.25 ± 1.83	14.42 ± 0.90	11.64 ± 1.20
GFAP content frontal cerebrum (μg GFAP/g protein)	0.64 ± 0.02	0.90 ± 0.13	0.70 ± 0.12
Frontal cerebrum magnesium (μg Mg^2+^/g brain tissue)	133.64 ± 4.97	135.59 ± 10.37	150.89 ± 5.23

All data are presented as mean ± SEM. Behavior and ventricle size are specified at by postnatal day (P) age. All brain structural and biochemical data are at P46

*CGalT* ceramide galactosyltransferase

*GFAP* glial fibrillary acidic protein

*GPC-PP* glycerylphosphorylcholine phosphocholine phosphodiesterase

*MBP* myelin basic protein

* p < 0.05 control vs. hydrocephalic, *t* tests or ANOVA

^@^ *p* < 0.05 NaCl vs. MgSO_4_-treated ferrets, *t* tests or ANOVA

^#^ *p* < 0.05 P29 (pre-treat) vs. P45 (post-treat), *t* tests

### Body weight and behavioral assessments

Behavioral testing began at P10–11 to determine baseline performance before kaolin injection was performed at P15. Successful induction of hydrocephalus was typically associated with weight loss for several days following injection. Overall, the hydrocephalic ferrets weighed 39 % less than nonhydrocephalic ferrets at P21 (*p* < 0.001, *t* test) and 25 % less at P28 (*p* = 0.009, *t* test). After the 14-day treatment period, the MgSO_4_-treated hydrocephalic ferrets weighed significantly less than the NaCl hydrocephalic group during and after treatment (all *p* < 0.05, ANOVA; Table 1).

By the end of the pretreatment period (P28–P30), all the ferrets were crawling, similar to previously assessed motor development [27]. Walking commenced on P34–37, with no appreciable differences between hydrocephalic groups. Qualitatively, both groups reached motor and behavioral developmental milestones at the same time, although several of the MgSO_4_-treated hydrocephalic ferrets (n = 5) were more unsteady and engaged in less exploratory behavior. Unlike our previous finding [27], the hydrocephalic ferrets did not become hyperactive or wander in circles within the enclosure. MgSO_4_-treated ferrets were more lethargic than NaCl-treated animals. Quantitative activity measurements showed that the hydrocephalic MgSO_4_ group displayed significantly less ambulatory movement, fewer cell entries, less distance traveled overall, and less supported rearing, compared to the NaCl group during the first week of treatment (all *p* < 0.05, ANOVA; data not shown). These between-group discrepancies were evident primarily during transition to walking phase of development but dissipated by the last behavioral time point measured and were not significantly different post-treatment (Table 1).

### Structural and biochemical changes in brain

**Fig. 3:**
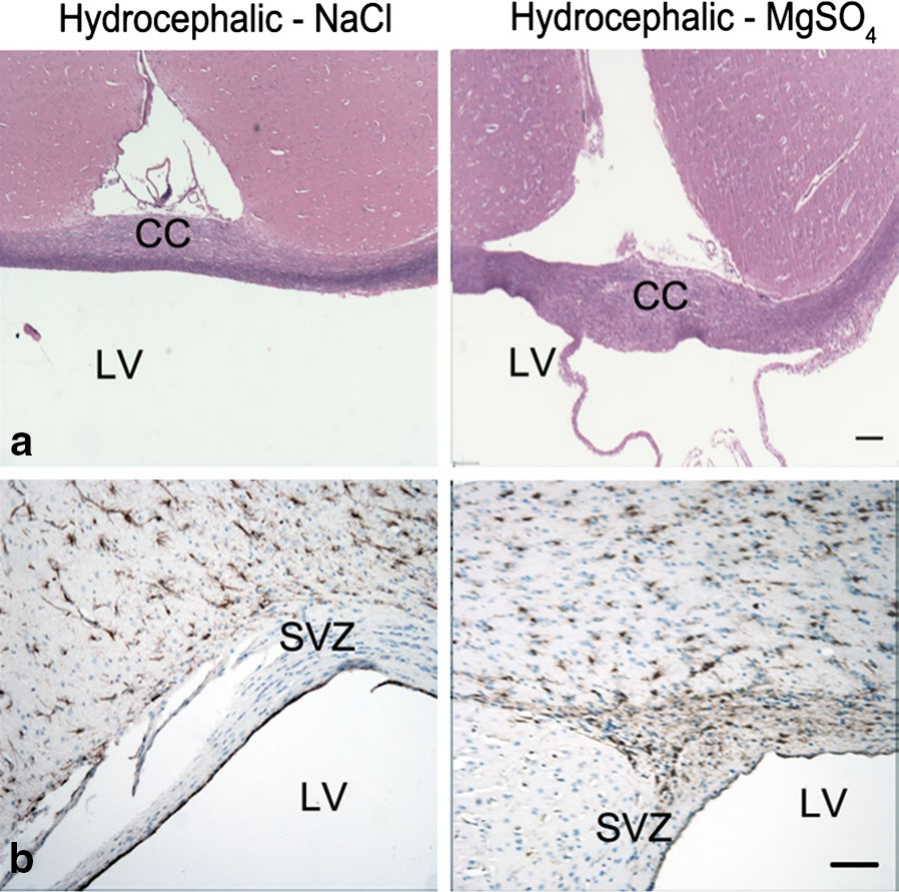
Photomicrographs showing white matter above the frontal horn of the lateral ventricle in 46-day-old hydrocephalic ferret brains, after a 14-day treatment period with MgSO_4_ or NaCl. **a** (*upper pair*)—corpus callosum *CC* thinning is evident in both animals (solochrome cyanine stains myelin *blue*, with *pink* eosin counterstain). **b** (*lower pair*)—GFAP immunolabeling (*brown*) showing hypertrophic reactive astrocytes in periventricular white matter near the lateral angle of the frontal horn. There is no obvious difference between the two groups of hydrocephalic ferrets. Objective magnification: **a**-40x, **b**-100x; *Scale bar* = 100 μm. Lateral ventricles *LV*, Subventricular zone SVZ

Macrophages that had engulfed kaolin and associated collagen deposition were microscopically identified throughout the basal subarachnoid space, having spread from the cisterna magna injection site; only rare macrophages were present in the fourth ventricle. Ventriculomegaly was accompanied by cerebral thinning surrounding the lateral ventricles and decreased depth of cerebral sulci. Intact white matter structures such as the internal capsule showed equivalent myelin staining in hydrocephalic and non-hydrocephalic ferrets. There was thinning and fraying of the periventricular white matter in all hydrocephalic ferrets; these damaged regions had no myelin staining. The corpus callosum thickness did not differ between the MgSO_4_ and NaCl treatment groups (Fig. 3; Table 1). In comparison to non-hydrocephalic ferrets, there was a slight but not statistically significant reduction of cerebral MBP content in the hydrocephalic ferrets; however, there was no difference between treatment groups (Table 1). To determine if the severity of ventriculomegaly had any impact on the MBP content, both treatment groups were subdivided into moderate and severe hydrocephalus. Subgroup analyses of the 4 most severely affected ferrets in each group showed no significant differences between groups (data not shown). It should be noted that the MBP content at P46 is just beginning the rapid accumulation phase [27]; this is comparable to human brain shortly after full term gestation.

In periventricular and perivascular foci of the white matter adjacent to the frontal horns of the lateral ventricles, NaCl and MgSO_4_-treated hydrocephalic ferrets displayed similar GFAP immunostaining of hypertrophic astrocytes (Fig. 3). ELISA analysis of GFAP content was 27 % lower in the MgSO_4_ hydrocephalic group compared to NaCl group, but the difference was not statistically significant (*p* = 0.375, ANOVA; Table 1). Subgroup analysis of the most severely affected ferrets also did not reveal significant differences in GFAP content between treatment groups (data not shown).

Myelin enzyme activity was measured in hydrocephalic ferret brains. CGalT is active in oligodendrocytes during myelin production [35]; we previously showed highest activity from P35–P56 [27]. There were no significant differences between hydrocephalic treatment groups (Table 1). GPC-PP is abundant in mature myelin [36]; we previously showed that the activity began to increase at P35 [27]. Compared to the untreated hydrocephalic ferrets, the MgSO_4_ group tended to show lower GPC-PP activity than the NaCl group (*p* = 0.056) (Table 1), but subgroup analysis still did not reach significance (data not shown).

Mg^2+^ in anterior forebrain samples was slightly, but not significantly, higher in the MgSO_4_ group compared to the NaCl-treated group (*p* = 0.38, *t* test) (Table 1).

## Discussion

Mg^2+^ was shown to have mild protective benefits in rats with experimental hydrocephalus treated from 5 to 7 weeks age but not in rats treated from 1 to 3 weeks age [25, 34]. We had hoped that MgSO_4_ treatment would also yield therapeutic benefits in young hydrocephalic ferrets. As has been recommended for preclinical stroke and brain trauma studies [26, 37], the experimental design included randomization, blinding, and multiple outcome measures. However, in comparison to NaCl-treated hydrocephalic ferrets, we did not find any behavioral, histological, or biochemical evidence to support the hypothesis that MgSO_4_ therapy, at the same dose that was effective in rats, benefits hydrocephalic ferrets. Treated ferrets had transient sedation, which is well-documented [38], impaired weight gain, and tendency to greater progression of ventriculomegaly. Despite more severely enlarged ventricles, MgSO_4_ treatment was associated with reduced GFAP accumulation in ferrets albeit not significantly; this finding is similar to that seen in hydrocephalic rats treated from 5 to 7 weeks age [25]. Reduced astroglial reaction has also been reported in kaolin-induced and congenitally hydrocephalic H-Tx rats treated with minocycline or decorin [39–41]. Although reduced GFAP accumulation is often considered an indicator of benefit, another possibility is that Mg^2+^, which blocks signaling between astrocytes [42], simply masks the astrocytic response to brain damage.

Why was MgSO_4_ therapy unsuccessful in hydrocephalic ferrets? Rationale for the experiment was based upon a previously-demonstrated neuroprotective effect in juvenile hydrocephalic rats and a range of experimental data from other neurological disorders. Technical and design aspects must be considered. It remains unclear whether entry of Mg^2+^ into brain is via the choroid plexus and CSF or through the blood brain barrier [43, 44], although the observed side effect of sedation indicates entry into the brain [8, 45, 46]. Lethargy is a potential confounder in the behavioral assessments, and it prevented complete blinding of the investigators. More importantly, weight gain was retarded in the MgSO_4_ treated ferrets, possibly because lethargy impaired feeding or because of the effect of Mg^2+^ on intestinal smooth muscle [7]. Undernutrition might have had a negative effect on the outcome. This might be overcome experimentally by using a matched feeding strategy. Unfortunately, ferrets are obligate carnivores with short intestinal tracts; they can require special diets when they are ill [47], and therefore might not be the ideal animal for studies where feeding is compromised. Periodic subcutaneous injections would result in troughs and peaks; in rats Mg^2+^ levels peak at approximately 2 h and return to normal levels within 4 h after injection [25]. We had considered using osmotic minipumps, but none would accommodate sufficiently large volumes for the treatment period nor could they adjust for the increasing weight of the maturing ferrets. Furthermore, based on discussions with the veterinarians, the potential complications (maternal biting of the surgical site and sloughing of the skin over the minipumps) outweighed the potential benefits. During the treatment period, we did observe that the MgSO_4_-treated ferrets experienced some skin irritation at the injection sites; rotating the locations of the injections minimized this. Oral administration potentially provides longer periods of exposure; however, peak levels are not as high as those that follow parenteral administration [48]. We also observed that some MgSO_4_-treated ferrets began gagging and/or vomiting immediately after injections, which would have made repeated gavage difficult. In a clinical situation, tube feeding would negate this confounder. Perhaps the intervention was timed incorrectly. If the ventricular enlargement is too mild a therapeutic benefit might be difficult to detect or if the intervention is too late, no benefit might be possible. However, subgroup analysis of the 4 most severely affected ferrets in each group as well as the least severely affected still showed no significant differences between treatment groups.

We must also consider that the rodent experimental studies considered in the Introduction do not translate to larger animals. The majority documented only short-term benefit. In short term studies of brain hypoxic-ischemic damage in immature rat, sheep, and pig models, MgSO_4_ has had inconsistent outcomes and results have been confounded by mild hypothermia; for this condition reviewers concluded “peripherally administered MgSO_4_ is unlikely to be neuroprotective” [49]. A meta-analysis of 8 randomized controlled trials with a total of 786 head-injured patients indicates that MgSO_4_ has no significant improvement for mortality, but there is borderline improvement in the glasgow outcome scale [50].

## Conclusion

Young hydrocephalic ferrets at the age during which cerebral myelin production is just beginning did not exhibit behavioral benefits or white matter protection from MgSO_4_ therapy. Although MgSO_4_ seems to be safe in other situations, it was associated with severe sedation, which can compromise feeding. Considering the experimental and epidemiologic evidence that Mg^2+^ might protect the developing brain in other diseases, this negative result should not completely exclude its possible benefits in hydrocephalus. However, preclinical testing might have to be done in a larger animal model wherein continuous delivery is possible or more control can be achieved over feeding. We will continue to explore other pharmacological agents that can be used as a supplement to shunt treatment of hydrocephalus in gyrencephalic animal models.
